# 2-Amino-5-bromo­pyridinium trifluoro­acetate

**DOI:** 10.1107/S1600536810008184

**Published:** 2010-03-06

**Authors:** Madhukar Hemamalini, Hoong-Kun Fun

**Affiliations:** a X-ray Crystallography Unit, School of Physics, Universiti Sains Malaysia, 11800 USM, Penang, Malaysia

## Abstract

In the title compound, C_5_H_6_BrN_2_^+^·C_2_F_3_O_2_^−^, the F atoms of the anion are disordered over two sets of sites, with occupancies of 0.59 (2):0.41 (2). In the crystal structure, the anions and cations are linked into a two-dimensional network parallel to (100) by N—H⋯O and C—H⋯O hydrogen bonds. Within this network, the N—H⋯O hydrogen bonds generate *R*_2_^2^(8) ring motifs.

## Related literature

For background to the chemistry of substituted pyridines, see: Pozharski *et al.* (1997 ▶); Katritzky *et al.* (1996 ▶). For related structures, see: Goubitz *et al.* (2001 ▶); Vaday & Foxman (1999 ▶). For details of hydrogen bonding, see: Jeffrey & Saenger (1991 ▶); Jeffrey (1997 ▶); Scheiner (1997 ▶). For hydrogen-bond motifs, see: Bernstein *et al.* (1995 ▶). For bond-length data, see: Allen *et al.* (1987 ▶).
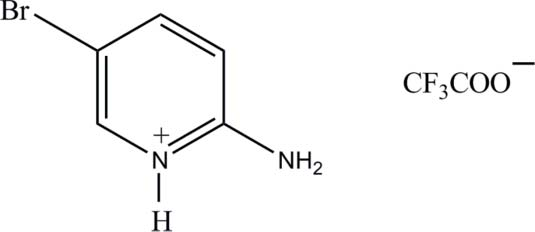


## Experimental

### 

#### Crystal data

C_5_H_6_BrN_2_^+^·C_2_F_3_O_2_^−^
*M* *_r_* = 287.05
Orthorhombic, 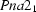

*a* = 17.5852 (13) Å
*b* = 11.3010 (9) Å
*c* = 5.1264 (4) Å
*V* = 1018.77 (14) Å^3^
*Z* = 4
Mo *K*α radiation
μ = 4.06 mm^−1^
*T* = 296 K
0.73 × 0.41 × 0.09 mm

#### Data collection

Bruker SMART APEXII CCD area-detector diffractometer
Absorption correction: multi-scan (*SADABS*; Bruker, 2009 ▶) *T*_min_ = 0.156, *T*_max_ = 0.709
8343 measured reflections
2899 independent reflections
1547 reflections with *I* > 2σ(*I*)
*R*_int_ = 0.056

#### Refinement

*R*[*F* ^2^ > 2σ(*F* ^2^)] = 0.039
*wR*(*F* ^2^) = 0.099
*S* = 0.93
2899 reflections
176 parameters
56 restraints
H atoms treated by a mixture of independent and constrained refinement
Δρ_max_ = 0.46 e Å^−3^
Δρ_min_ = −0.28 e Å^−3^
Absolute structure: Flack (1983 ▶), 1254 Friedel pairs
Flack parameter: 0.024 (12)

### 

Data collection: *APEX2* (Bruker, 2009 ▶); cell refinement: *SAINT* (Bruker, 2009 ▶); data reduction: *SAINT*; program(s) used to solve structure: *SHELXTL* (Sheldrick, 2008 ▶); program(s) used to refine structure: *SHELXTL*; molecular graphics: *SHELXTL*; software used to prepare material for publication: *SHELXTL* and *PLATON* (Spek, 2009 ▶).

## Supplementary Material

Crystal structure: contains datablocks global, I. DOI: 10.1107/S1600536810008184/ci5042sup1.cif

Structure factors: contains datablocks I. DOI: 10.1107/S1600536810008184/ci5042Isup2.hkl

Additional supplementary materials:  crystallographic information; 3D view; checkCIF report

## Figures and Tables

**Table 1:** Hydrogen-bond geometry (Å, °)

*D*—H⋯*A*	*D*—H	H⋯*A*	*D*⋯*A*	*D*—H⋯*A*
N1—H1*N*1⋯O1^i^	0.93 (3)	1.80 (3)	2.720 (5)	171 (3)
N2—H1*N*2⋯O1^ii^	0.83 (4)	2.05 (4)	2.870 (4)	176 (5)
N2—H2*N*2⋯O2^i^	0.83 (4)	2.03 (4)	2.849 (5)	170 (4)
C1—H1*A*⋯O2^iii^	0.93	2.34	3.245 (4)	164

Symmetry codes: (i) 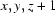; (ii) 
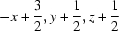
; (iii) 
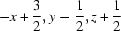
.

**Fig. 1.**
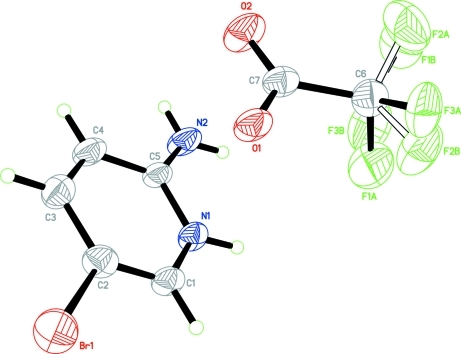
The asymmetric unit of the title compound. Displacement ellipsoids are drawn at the 30% probability level. Both disorder components are shown.

**Fig. 2.**
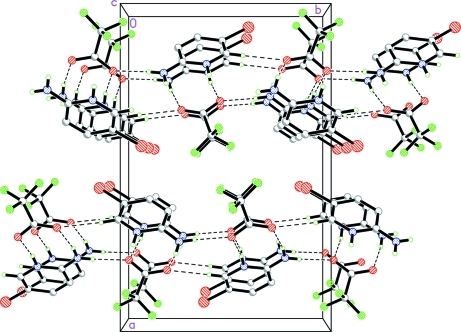
The crystal packing of the title compound, showing hydrogen-bonded (dashed lines) networks.

## supplementary crystallographic information

### Comment

Pyridine and its derivatives play an important role in heterocyclic chemistry (Pozharski *et al.*, 1997; Katritzky *et al.*, 1996). They are often involved in hydrogen-bond interactions (Jeffrey & Saenger, 1991; Jeffrey, 1997; Scheiner, 1997). The crystal structures of 2-amino-5-bromopyridine (Goubitz *et al.*, 2001) and 2-amino-5-bromopyridinium propynoate (Vaday & Foxman, 1999) have been reported. In order to study hydrogen bonding interactions, the title salt was prepared and its crystal structure is reported here.

The asymmetric unit of (I) (Fig. 1) contains one 2-amino-5-bromopyridinium cation and one trifluoroacetate anion, indicating that proton transfer has occurred during the co-crystallisation. The 2-amino-5-methylpyridinium cation is essentially planar,  with a maximum deviation of 0.016 (4) Å for atom C1; As a result of protonation, the C1—N1—C5 angle is widened to 122.5 (4)°. The bond lengths and angles are normal (Allen *et al.*, 1987).

In the crystal packing (Fig. 2), the cations and anions are linked via N—H···O hydrogen bonds to form *R*_2_^2^(8) ring motifs (Bernstein *et al.*, 1995). The ionic units are linked into a two-dimensional network parallel to the (100) by N—H···O and C—H···O hydrogen bonds (Table 1).

### Experimental

To a hot methanol solution (20 ml) of 2-amino-5-bromopyridine (44 mg, Aldrich) was added a few drops of trifluoroacetic acid.  The solution was warmed over a water bath for a few minutes. The resulting solution was allowed to cool slowly to room temperature. Crystals of the title compound appeared after a few days.

### Refinement

Atoms H1N1, H1N2 and H2N2 were located in a difference Fourier map and refined; the N–H distances of the NH_2_ group were restrained to be equal. The remaining H atoms were positioned geometrically [C–H = 0.93 Å] and were refined using a riding model, with *U*_iso_(H) = 1.2*U*_eq_(C). The F atoms of the anion are disordered over two positions with occupancies of 0.59 (2):0.41 (2). The C—F distances were restrained to be equal and the U^ij^ components of F atoms were restrained to an approximate isotropic behaviour.

### Crystal data

**Table:**

C_5_H_6_BrN_2_^+^·C_2_F_3_O_2_^−^	*F*(000) = 560
*M**_r_* = 287.05	*D*_x_ = 1.871 Mg m^−^^3^
Orthorhombic, *P**n**a*2_1_	Mo *K*α radiation, λ = 0.71073 Å
Hall symbol: P 2c -2n	Cell parameters from 1994 reflections
*a* = 17.5852 (13) Å	θ = 2.9–22.5°
*b* = 11.3010 (9) Å	µ = 4.06 mm^−^^1^
*c* = 5.1264 (4) Å	*T* = 296 K
*V* = 1018.77 (14) Å^3^	Plate, colourless
*Z* = 4	0.73 × 0.41 × 0.09 mm

### Data collection

**Table:**

Bruker SMART APEXII CCD area-detector diffractometer	2899 independent reflections
Radiation source: fine-focus sealed tube	1547 reflections with *I* > 2σ(*I*)
graphite	*R*_int_ = 0.056
φ and ω scans	θ_max_ = 30.1°, θ_min_ = 2.1°
Absorption correction: multi-scan (*SADABS*; Bruker, 2009)	*h* = −24→24
*T*_min_ = 0.156, *T*_max_ = 0.709	*k* = −14→15
8343 measured reflections	*l* = −7→7

### Refinement

**Table:**

Refinement on *F*^2^	Secondary atom site location: difference Fourier map
Least-squares matrix: full	Hydrogen site location: inferred from neighbouring sites
*R*[*F*^2^ > 2σ(*F*^2^)] = 0.039	H atoms treated by a mixture of independent and constrained refinement
*wR*(*F*^2^) = 0.099	*w* = 1/[σ^2^(*F*_o_^2^) + (0.0247*P*)^2^] where *P* = (*F*_o_^2^ + 2*F*_c_^2^)/3
*S* = 0.93	(Δ/σ)_max_ = 0.001
2899 reflections	Δρ_max_ = 0.46 e Å^−^^3^
176 parameters	Δρ_min_ = −0.28 e Å^−^^3^
56 restraints	Absolute structure: Flack (1983), 1254 Friedel pairs
Primary atom site location: structure-invariant direct methods	Flack parameter: 0.024 (12)

### Special details

**Table:**

Geometry. All s.u.'s (except the s.u. in the dihedral angle between two l.s. planes) are estimated using the full covariance matrix. The cell s.u.'s are taken into account individually in the estimation of s.u.'s in distances, angles and torsion angles; correlations between s.u.'s in cell parameters are only used when they are defined by crystal symmetry. An approximate (isotropic) treatment of cell s.u.'s is used for estimating s.u.'s involving l.s. planes.
Refinement. Refinement of F^2^ against ALL reflections. The weighted R-factor wR and goodness of fit S are based on F^2^, conventional R-factors R are based on F, with F set to zero for negative F^2^. The threshold expression of F^2^ > 2σ(F^2^) is used only for calculating R-factors(gt) etc. and is not relevant to the choice of reflections for refinement. R-factors based on F^2^ are statistically about twice as large as those based on F, and R- factors based on ALL data will be even larger.

### Fractional atomic coordinates and isotropic or equivalent isotropic displacement parameters (Å^2^)

**Table:**

	*x*	*y*	*z*	*U*_iso_*/*U*_eq_	Occ. (<1)
Br1	0.93625 (2)	0.38997 (4)	0.26064 (12)	0.0770 (2)	
N1	0.80785 (16)	0.5888 (2)	0.7575 (9)	0.0462 (6)	
N2	0.7800 (2)	0.7862 (3)	0.8112 (8)	0.0631 (11)	
C1	0.8432 (2)	0.4973 (3)	0.6357 (8)	0.0489 (8)	
H1A	0.8355	0.4204	0.6947	0.059*	
C2	0.8889 (2)	0.5166 (4)	0.4322 (8)	0.0540 (9)	
C3	0.9016 (2)	0.6353 (4)	0.3492 (8)	0.0585 (11)	
H3A	0.9342	0.6507	0.2106	0.070*	
C4	0.8661 (2)	0.7253 (4)	0.4722 (8)	0.0563 (10)	
H4A	0.8743	0.8029	0.4183	0.068*	
C5	0.81681 (19)	0.7018 (3)	0.6821 (7)	0.0474 (9)	
O1	0.70895 (14)	0.5277 (2)	0.1425 (6)	0.0589 (7)	
O2	0.67948 (17)	0.7148 (2)	0.2175 (8)	0.0751 (9)	
F1A	0.6204 (5)	0.5331 (17)	0.6225 (16)	0.120 (4)	0.59 (2)
F2A	0.5460 (5)	0.6483 (7)	0.419 (3)	0.104 (3)	0.59 (2)
F3A	0.5673 (5)	0.4758 (8)	0.273 (3)	0.095 (3)	0.59 (2)
F1B	0.5407 (6)	0.6290 (16)	0.294 (4)	0.115 (5)	0.41 (2)
F2B	0.5819 (10)	0.4561 (7)	0.392 (4)	0.105 (5)	0.41 (2)
F3B	0.6028 (10)	0.6030 (19)	0.6312 (19)	0.113 (5)	0.41 (2)
C6	0.5992 (2)	0.5688 (3)	0.3866 (8)	0.0636 (11)	
C7	0.66887 (19)	0.6091 (3)	0.2336 (9)	0.0510 (9)	
H1N1	0.7730 (19)	0.576 (3)	0.891 (7)	0.041 (9)*	
H1N2	0.785 (2)	0.856 (3)	0.767 (11)	0.060 (11)*	
H2N2	0.748 (2)	0.773 (4)	0.927 (7)	0.067 (14)*	

### Atomic displacement parameters (Å^2^)

**Table:**

	*U*^11^	*U*^22^	*U*^33^	*U*^12^	*U*^13^	*U*^23^
Br1	0.0861 (3)	0.0606 (3)	0.0842 (3)	0.0050 (2)	0.0152 (3)	−0.0136 (3)
N1	0.0546 (15)	0.0281 (14)	0.0561 (15)	−0.0047 (11)	0.002 (2)	−0.004 (2)
N2	0.085 (2)	0.0291 (17)	0.075 (3)	0.0045 (18)	0.014 (2)	0.0079 (18)
C1	0.058 (2)	0.0280 (18)	0.061 (2)	−0.0045 (16)	−0.0053 (18)	−0.0018 (17)
C2	0.059 (2)	0.040 (2)	0.063 (2)	0.0022 (17)	−0.0031 (18)	−0.0071 (18)
C3	0.061 (2)	0.054 (3)	0.061 (3)	−0.007 (2)	0.0016 (18)	0.0057 (19)
C4	0.067 (2)	0.037 (2)	0.064 (2)	−0.0068 (19)	−0.004 (2)	0.013 (2)
C5	0.0519 (18)	0.0322 (19)	0.058 (2)	−0.0045 (16)	−0.0059 (16)	0.0088 (16)
O1	0.0679 (15)	0.0319 (14)	0.0768 (16)	0.0077 (12)	0.0135 (14)	0.0109 (13)
O2	0.0987 (19)	0.0303 (14)	0.096 (3)	0.0012 (13)	0.0185 (18)	0.0044 (18)
F1A	0.115 (5)	0.166 (9)	0.078 (4)	−0.013 (5)	0.011 (3)	0.046 (5)
F2A	0.093 (4)	0.080 (4)	0.139 (8)	0.018 (3)	0.040 (4)	−0.019 (4)
F3A	0.083 (3)	0.082 (4)	0.119 (6)	−0.036 (3)	0.008 (4)	−0.011 (5)
F1B	0.069 (5)	0.143 (8)	0.132 (9)	0.030 (5)	0.009 (6)	−0.004 (7)
F2B	0.119 (7)	0.062 (6)	0.133 (9)	−0.011 (5)	0.047 (7)	−0.001 (5)
F3B	0.130 (8)	0.121 (9)	0.088 (6)	−0.024 (6)	0.036 (5)	−0.009 (6)
C6	0.065 (3)	0.045 (2)	0.080 (3)	0.002 (2)	0.005 (2)	−0.007 (2)
C7	0.062 (2)	0.037 (2)	0.054 (2)	0.0022 (16)	−0.005 (2)	0.001 (2)

### Geometric parameters (Å, °)

**Table:**

Br1—C2	1.875 (4)	C4—C5	1.407 (5)
N1—C5	1.344 (4)	C4—H4A	0.93
N1—C1	1.358 (5)	O1—C7	1.250 (4)
N1—H1N1	0.93 (4)	O2—C7	1.212 (4)
N2—C5	1.330 (5)	F1A—C6	1.329 (6)
N2—H1N2	0.82 (3)	F2A—C6	1.307 (6)
N2—H2N2	0.84 (3)	F3A—C6	1.327 (6)
C1—C2	1.334 (5)	F1B—C6	1.321 (7)
C1—H1A	0.93	F2B—C6	1.310 (7)
C2—C3	1.425 (6)	F3B—C6	1.314 (7)
C3—C4	1.349 (6)	C6—C7	1.524 (6)
C3—H3A	0.93		
			
C5—N1—C1	122.5 (4)	N2—C5—N1	118.8 (3)
C5—N1—H1N1	116 (2)	N2—C5—C4	123.0 (4)
C1—N1—H1N1	121 (2)	N1—C5—C4	118.1 (4)
C5—N2—H1N2	120 (4)	F2B—C6—F3B	106.2 (8)
C5—N2—H2N2	124 (3)	F2B—C6—F1B	109.0 (10)
H1N2—N2—H2N2	116 (5)	F3B—C6—F1B	103.2 (9)
C2—C1—N1	120.7 (4)	F2A—C6—F3A	107.3 (7)
C2—C1—H1A	119.6	F2A—C6—F1A	107.2 (6)
N1—C1—H1A	119.6	F3A—C6—F1A	106.1 (6)
C1—C2—C3	118.8 (4)	F2A—C6—C7	115.8 (6)
C1—C2—Br1	120.6 (3)	F2B—C6—C7	119.1 (7)
C3—C2—Br1	120.6 (3)	F3B—C6—C7	111.4 (7)
C4—C3—C2	119.8 (4)	F1B—C6—C7	106.8 (8)
C4—C3—H3A	120.1	F3A—C6—C7	110.4 (6)
C2—C3—H3A	120.1	F1A—C6—C7	109.5 (5)
C3—C4—C5	120.0 (4)	O2—C7—O1	127.8 (4)
C3—C4—H4A	120.0	O2—C7—C6	116.9 (4)
C5—C4—H4A	120.0	O1—C7—C6	115.2 (3)
			
C5—N1—C1—C2	−0.2 (6)	F2B—C6—C7—O2	174.5 (14)
N1—C1—C2—C3	1.7 (5)	F3B—C6—C7—O2	−61.4 (13)
N1—C1—C2—Br1	−178.9 (3)	F1B—C6—C7—O2	50.6 (12)
C1—C2—C3—C4	−1.6 (6)	F3A—C6—C7—O2	142.0 (8)
Br1—C2—C3—C4	179.0 (3)	F1A—C6—C7—O2	−101.5 (10)
C2—C3—C4—C5	0.1 (6)	F2A—C6—C7—O1	−161.2 (9)
C1—N1—C5—N2	179.8 (3)	F2B—C6—C7—O1	−6.5 (14)
C1—N1—C5—C4	−1.4 (6)	F3B—C6—C7—O1	117.6 (12)
C3—C4—C5—N2	−179.8 (4)	F1B—C6—C7—O1	−130.4 (12)
C3—C4—C5—N1	1.4 (5)	F3A—C6—C7—O1	−39.0 (8)
F2A—C6—C7—O2	19.8 (10)	F1A—C6—C7—O1	77.5 (10)

### Hydrogen-bond geometry (Å, °)

**Table:**

*D*—H···*A*	*D*—H	H···*A*	*D*···*A*	*D*—H···*A*
N1—H1N1···O1^i^	0.93 (3)	1.80 (3)	2.720 (5)	171 (3)
N2—H1N2···O1^ii^	0.83 (4)	2.05 (4)	2.870 (4)	176 (5)
N2—H2N2···O2^i^	0.83 (4)	2.03 (4)	2.849 (5)	170 (4)
C1—H1A···O2^iii^	0.93	2.34	3.245 (4)	164

Symmetry codes: (i) *x*, *y*, *z*+1; (ii) −*x*+3/2, *y*+1/2, *z*+1/2; (iii) −*x*+3/2, *y*−1/2, *z*+1/2.
